# Evaluation of Carcass Attributes and Physical, Chemical, and Qualitative Characteristics of Breast Meat of Broiler Chickens Fed on *Pulicaria jaubertii* Powder

**DOI:** 10.3390/life13081780

**Published:** 2023-08-21

**Authors:** Hani H. Al-Baadani, Ibrahim A. Alhidary, Abdulrahman S. Alharthi, Mahmoud M. Azzam, Gamaleldin M. Suliman, Mohammed A. Ahmed, Akram A. Qasem

**Affiliations:** 1 Department of Animal Production, College of Food and Agriculture Science, King Saud University, P.O. Box 2460, Riyadh 11451, Saudi Arabia; ialhidary@ksu.edu.sa (I.A.A.); abalharthi@ksu.edu.sa (A.S.A.); mazzam@ksu.edu.sa (M.M.A.); gsuliman@ksu.edu.sa (G.M.S.); 2 Department of Food Science and Nutrition, College of Food and Agriculture Science, King Saud University, P.O. Box 2460, Riyadh 11451, Saudi Arabia; masifa@ksu.edu.sa (M.A.A.); aqasem@ksu.edu.sa (A.A.Q.)

**Keywords:** broilers, carcass, meat quality, performance, *Pulicaria jaubertii*

## Abstract

*Pulicaria jaubertii* (PJ) is a medicinal plant used as a synthetic antioxidant and as a traditional medicine due to its bioactive compounds. The objective of this study was to investigate the effects of PJ on carcass traits and breast meat quality parameters of broiler chickens. Two hundred and forty male broilers (1 day old) were divided into four groups (0, 3, 6, and 9 g of PJ/kg of basal diet). Performance indicators were evaluated during the feeding stages, and carcass characteristics and physiochemical and qualitative parameters of breast meat were measured at 36 days old. The results showed that PJ improved performance parameters such as weight gain, feed conversion ratio, and production efficiency index (*p* < 0.05) in the finishing stage. The diets supplemented with PJ were associated with better carcass characteristics (*p* < 0.05), but some body parts, such as legs (6 and 9 g PJ) and backs (3–9 g PJ) decreased (*p* < 0.05). Temperature and initial pH were decreased by PJ (*p* < 0.05). Meat color was not affected by PJ (*p* > 0.05), although the yellowness and saturation index were lower at 9 g PJ. Total saturated fatty acid content was higher at 3 g PJ, while total polyunsaturated fatty acids and unsaturated to saturated fatty acid ratio were lower at 3 and 6 g PJ (*p* < 0.05). Total monounsaturated fatty acid content increased at 6 and 9 g PJ. Omega-6 fatty acids and the ratio of omega-6 to omega-3 were lower at 3 g PJ. PJ resulted in higher weight loss on cooking (6 and 9 g PJ) and shear force (3–9 g PJ). In conclusion, PJ had a positive influence on performance, carcass characteristics, and fatty acid profile, and some meat quality traits were generally improved by PJ, but knowledge of its mode of action is still limited and therefore requires further investigation.

## 1. Introduction

Currently, there is an increasing interest in modern broiler chicken production, focusing on nutrition and nutritional supplementation to produce a greater quantity of meat, provided it is of high quality and safe for human consumption [1,2]. However, chicken meat is considered an economically appropriate food compared to other types of animal products and is accepted by all cultures and religions [3,4]. Moreover, it is characterized by easy digestibility, high protein and low fat content, and high polyunsaturated fatty acids content, which has increased in importance in recent years [5,6]. Therefore, paying attention to feed quality is one of the basic requirements for promoting growth and improving meat quality by meeting the physiological needs of broilers [7]. On the other hand, after the ban on the use of antibiotics in broiler feeds, the use of dietary supplements, including medicinal plants, has become a safe alternative for feed additives due to the increasing consumer demand for antibiotic-free poultry products, the development of bacterial resistance, and increasing consumer concern, which has led to numerous studies on the discovery of dietary supplements from aromatic medicinal plants in recent years [8,9,10].

*Pulicaria jaubertii* (PJ), considered an aromatic medicinal plant, belongs to the Asteraceae family and is widely distributed as a wild plant in Yemen and Saudi Arabia [11,12]. Traditionally, PJ is used as a spice and as a safe substitute for synthetic antioxidants to protect food [13,14,15]. A study by Alharthi et al. [16] found that the whole plant of PJ (stems, leaves, and flowers) contains proteins; minerals; fiber; saturated and unsaturated fatty acids; and bioactive compounds, such as flavonoids, phenols, and terpenoids. Several previous studies have been conducted with extracts of PJ, as they were found to have antioxidant properties due to their bioactive compounds [17,18]. The use of other aromatic plants as natural dietary supplements, such as *Pulicaria gnaphalodes* powder [19], *Pulicaria undulata* powder [20], and *Eucalyptus globulus* oil [21], has demonstrated that these bioactive compounds can enhance the performance and meat quality of broiler chickens. 

To the best of our knowledge, no previous studies have investigated the use of PJ as a natural feed additive and its effects on carcass characteristics and breast meat quality of broiler chickens. The present study may provide evidence for future applications of PJ powder as a feed additive to improve broiler chicken performance and meat quality, which is the basis of the hypothesis of this study. Therefore, the aim of this study is to investigate the effect of PJ, as an aromatic medicinal plant, on performance, carcass characteristics, and breast meat quality in broilers.

## 2. Materials and Methods

### 2.1. Ethical Approval

All methods and procedures used in this study were approved by the Scientific Research Ethics Committee of King Saud University (KSU-SE-21-47).

### 2.2. Preparation of PJ Powder

PJ was collected directly as a whole plant (stems, leaves, and flowers) from the valleys around Ibb City, Republic of Yemen. It was then identified in the Kingdom of Saudi Arabia by the Herbarium of the College of Science at KSU, as it is considered a wild plant in southern Saudi Arabia (classification reference: 24544); dried at room temperature; and ground into a fine powder.

### 2.3. Chicks, Housing, and Study Design

A total of 240 1-day-old male Ross 308 broiler chicks were used. Each chick was weighed; then, the chicks were randomly divided into four levels of PJ powder (one kilogram of basal diet as control group supplemented with 0, 3, 6, or 9 g of PJ powder as whole plant) with ten replicate cages per level and six chicks per cage. The Ross 308 Management Guide (Aviagen, 2019, New York, NY, USA) recommends three feeding stages for the basal diet: starter (1–10 days old), growth (11–24 days old), and finisher (25–36 day old). The basal diet was prepared as mash to meet nutrient requirements during the study in accordance with these guidelines (see Table 1). The current study was conducted in a caged chamber under optimal growth conditions in accordance with the requirements of Ross 308 chickens, with temperature and humidity initially set at 33 °C and 50%, respectively, then gradually reduced (2 °C every three days) to 22 °C and 50% after 27 days. Up to seven days of age, the lighting program was available for 24 h (30–40 lux), then for 23 h (minimum 20 lux). Unlimited amounts of feed and water were available to the birds throughout the study. According to the recommendations, all birds received immunizations against Newcastle disease, infectious bronchitis (at 5 and 22 days old), and infectious bursal disease (at 14 days old).

### 2.4. Growth Performance

In three feeding stages (10, 24, and 36 days old), live body weight and feed consumption were recorded to calculate the weight gain (final body weight − initial body weight), feed intake (feed provided-residual feed), and feed conversion ratio (feed intake/body weight gain) according to Diler et al. [22]. Furthermore, the production efficiency index was calculated according to Biesek et al. [23] using the following equation: ((100 × body weight)/(age × feed conversion ratio)/10).

### 2.5. Carcass Traits

At the finisher stage (36 days old), all broilers were fasted overnight. Ten chickens were randomly selected for slaughter from each PJ level at a rate of one chicken per cage (total = 40). According to the method of Liu et al. [24], the preslaughter, the hot carcass (15 min post slaughter), the cold carcass (at 24 h post slaughter), and the body components (legs, breast, back, and abdominal fat) were weighed. Dressing yield (hot carcass weight/preslaughter body weight × 100) was calculated [25]. The body component weight was calculated as a percentage according to hot weight [26].

### 2.6. Physiochemical Properties of Breast Meat

pH and temperature were measured initially and after slaughter (15 min and 24 h, respectively) using a pH meter (Hanna Instruments, 211, Smithfield, RI, USA), and color (lightness, redness, and yellowness) was measured using a Minolta Chroma Meter (Konica Minolta, CR -400-Japan, Tokyo, Japan) according to Tashla et al. [27]. The approaches of Valizadeh et al. [28] and Adeyemi [29] were used to calculate color change (Sqrt (lightness − 94.18)^2^ + (redness − 0.43)^2^ + (yellowness − 3.98)^2^), saturation index (Sqrt (redness)^2^ + (yellowness)^2^), hue angle (tan − (yellowness/redness)), and browning index ((100 × (χ − 0.031)/0.17), where χ = redness + (1.75 × lightness)/(5.645 × lightness) + redness − (3.012 × yellowness)) using the color measurements. The chemical properties of breast meat (10 samples from each PJ level), including moisture, protein, fat, ash, and organic matter (100 − ash) were determined [30]. The total amounts of saturated, monounsaturated, polyunsaturated, omega-6, and omega-3 fatty acids were analyzed by gas chromatography–mass spectrometry (Agilent Technologies, Palo Alto, CA, USA) and expressed as a percentage based on total fatty acid methyl esters using a method previously described in [31,32]. Analysis of physical and chemical properties was performed in triplicate for each sample.

### 2.7. Breast Meat Quality

Ten samples of breast meat from each PJ level were analyzed after being cooked in an electric oven until an internal temperature of 70 °C was reached for measurement of cooking loss (difference between sample weight before and after cooking multiplied by 100) and shear force (tenderness, kg/cm^2^) and texture profile analysis according to Lee et al. [33] using a TA-HD Texture Analyzer with Warner–Bratzler shear barb (Stable Micro Systems Ltd., Godalming, UK). Water-holding capacity was determined by the filter paper press method and expressed as a percentage based on a 100 − (sample weight after water loss divided by sample weight multiplied by 100) [33].

### 2.8. Statistical Analysis

Statistical Analysis System software [34] was used. All data were statistically analyzed using one-way analysis of variance with a completely randomized design. The following statistical model was used:

Observed values (Yij)=general mean+PJ levels (PJi)+random error(eij)

The normality of the data (skewness, kurtosis, and box plot) was tested. Duncan’s multiple range test was used to analyze differences between means of PJ levels at *p* < 0.05. Data for each parameter are expressed as mean ± standard error of the mean.

## 3. Results

### 3.1. Performance Indicators

The effects of PJ levels on broiler performance indicators are shown in Table 2. The study findings demonstrate that broiler diets supplemented with 3 to 9 g PJ/kg of basal diet had no effect (*p* > 0.05) on the parameters of growth performance, except for body weight and production efficiency index, which were higher (*p* < 0.05) in the diet supplemented with 3-9 g and 3 g PJ/kg, respectively, compared with the non-supplemented basal diet (0 g PJ/kg) during the starter phase of 1 to 10 days old. In the grower phase (11–24 days old), PJ levels had no effect on body weight gain, feed intake, or production efficiency index (*p* > 0.05), whereas the feed conversion ratio improved when the diet was supplement with 9 g PJ/kg compared with non-supplemented basal diet (*p* < 0.05) but was not significantly different from the other levels (3 and 6 g PJ/kg). At 25–36 days old (finisher stage), performance indicators (body weight, weight gain, and production efficiency index) increased, and the feed conversion ratio improved under a 3–9 PJ/kg basal diet compared with a non-supplemented basal diet (*p* < 0.05), while PJ levels had no effect on feed intake (*p* > 0.05).

### 3.2. Carcass Traits

The effects of PJ levels on carcass traits and body components of broilers are shown in Table 3. Diets supplemented with PJ (3 to 9 g PJ/kg of the basal diet) were associated with higher preslaughter, hot carcass, and cold carcass weights compared to a non-supplemented basal diet (*p* < 0.05). Diets supplemented with 3 to 9 g PJ had no effect on relative weights of dressing yield and breast compared with the non-supplemented basal diet (*p* > 0.05). Relative leg weight decreased at levels of 6 and 9 g PJ, and relative back weight was also lower at 3 to 9 g PJ compared with a non-supplemented basal diet (*p* < 0.05). The 9 g PJ/kg basal diet was associated with lower abdominal fat compared with 3 g PJ (*p* < 0.05).

### 3.3. Physical Indicators of Breast Meat

The effects of PJ levels on the physical properties of broiler breast meat are shown in Table 4. Diets supplemented with PJ (3 to 9 g PJ/kg of the basal diet) were associated with lower temperature and pH values 15 min after slaughter than the non-supplemented basal diet (*p* < 0.05). The diet supplemented with 9 g PJ/kg of the basal diet had a lower ultimate pH (*p* < 0.05), but the diet supplemented with 3 g PJ/kg had a lower pH difference between initial and ultimate compared with the other supplements (*p* < 0.05). Breast meat color was not affected by PJ either initially or ultimately (*p* > 0.05), except 24 h after slaughter (ultimate), at which point yellowness and the saturation index were lower in broilers fed 9 g PJ/kg compared with broilers on a non-supplemented basal diet (*p* < 0.05).

### 3.4. Breast Meat Chemical Indices

The effects of PJ levels on the chemical properties of broiler breast meat are shown in Table 5. Diets supplemented with 3 to 9 g PJ had no effect on moisture, crude protein, crude fat, ash, or organic matter content compared with the non-supplemented basal diet (*p* > 0.05). The results also showed that the individual fatty acids, such as capric acid, lauric acid, palmitic acid, heptadecanoic acid, stearic acid, pristanic acid, lignoceric acid, myristic acid, pentadecanoic acid, tetradecenoic acid, myristioleic acid, palmitoleic acid, vaccenic acid, oleic acid, linoleic acid, arachidonic acid, and linolenic acid, were not significantly different between diets supplemented with 3 to 9 g PJ (*p* > 0.05; see Table S1). On the other hand, the results showed that broiler diets supplemented with 3 g PJ/kg of the basal diet had higher total saturated fatty acids, while the total polyunsaturated fatty acids and the ratio between total unsaturated and saturated fatty acids were lower at levels of 3 and 6 g PJ /kg compared with a non-supplemented basal diet (*p* < 0.05). Total monounsaturated fatty acids increased at levels of 3 and 9 g PJ compared with a non-supplemented basal diet (*p* < 0.05). Omega-6 fatty acids and the ratio of omega-6 to omega-3 decreased at levels of 3 g PJ compared with a non-supplemented basal diet (*p* < 0.05).

### 3.5. Breast Meat Quality Indices

The effects of PJ levels on broiler breast meat quality are shown in Table 6. Diets supplemented with 3 to 9 g PJ had no effect on the percentage of water-holding capacity or any of the parameters of texture profile analysis compared with the non-supplemented basal diet (*p* > 0.05). On the other hand, the results show that broiler diets supplemented with PJ were associated with higher cooking water loss percentages (6 and 9 g PJ/kg) and shear force values (3 to 9 g PJ/kg) compared to non-supplemented basal diet (*p* < 0.05).

## 4. Discussion

Aromatic medicinal plants have been used since ancient times, but they are still used today in the pharmaceutical industry, for traditional treatments, and as dietary supplements [35]. These plants contain many bioactive compounds that are part of regular metabolic processes, such as phenols, flavonoids, alkaloids, and saponins, among others, are also called phytochemicals [36,37]. PJ is an aromatic medicinal plant used as a spice, as a safe substitute for synthetic antioxidants, and for traditional treatment of humans [13,14,15]. It is rich in bioactive compounds such as flavonoids, terpenoids, and phenols, which can improve the performance and meat quality of broiler chickens [19,20]. The results obtained in this study show that production efficiency, as an indicator of economic performance, health, and welfare status of broiler chickens, was higher in diets supplemented with PJ powder, especially at 3 g PJ in the starter phase (1 to 10 days old), whereas dietary supplementation of 9 g PJ during the grower phase (11 to 24 days old) improved feed conversion compared to a non-supplemented basal diet but had no effect on other parameters of growth performance. In addition, supplementation with PJ powder (3 to 9 PJ/kg of the basal diet) in the finisher phase (25 to 36 days old) had a significant effect, increasing body weight gain and production efficiency index and improving the feed conversion ratio. These results may be due to the properties of the bioactive chemical compounds contained in the PJ powder, which are consistent with the results of previous studies that reported that phenols, flavonoids, and fatty acid compounds from aromatic medicinal plants can enhance growth performance by stimulating the secretion of endogenous intestinal enzymes and improving nutrient digestion and intestinal morphology in broiler chickens [16,38,39]. However, some aromatic plants, such as peppermint [40], ginger, and turmeric powder [41], can be used in broiler diets as growth promoters to enhance performance. In addition, growth performance, including live weight, weight gain, and feed conversion ratio, was improved in broiler chickens by a diet supplemented with 3 g of *Pulicaria undulata* (a species of the genus Pulicaria) [20]. No studies have been conducted to date on the use of PJ in broilers, but the present study may provide evidence for future applications to improve performance. Diets supplemented with PJ powder resulted in improved carcass characteristics (preslaughter weight, hot carcass weight, and cold carcass weight) and did not affect the relative weight of dressing yield compared to the basal diet. This suggests that the use of PJ powder as a feed additive could improve growth performance and carcass characteristics due to its chemical properties and bioactive compound content. The high weights of hot and cold carcasses due to the addition of PJ could be related to the final body weight before slaughter. This is consistent with the results of Ali [42] and Tashla et al. [27], who found that different levels of aromatic medicinal plant powder effectively improved the carcass characteristics of broiler chickens. In addition, aromatic medicinal plant powders (cinnamon twigs and star anise fruit) did not negatively affect performance and carcass traits [43]. Diets supplemented with levels of PJ powder were associated with lower relative weights of legs (3–9 g PJ), back (3 and 9 g PJ), and abdominal fat (9 g PJ) compared to unsupplemented basal diets, but this may be due to bioactive compounds in the PJ powder, which may inhibit lipogenesis. This is consistent with previous findings that higher levels of PJ lower serum triglyceride concentrations [16].

Indicators of physical properties of chicken breast meat, including pH and color, may play a critical role in determining the quality of fresh meat at the time of sale [44]. The maintenance of pH within the range after slaughter (initial and ultimate) is important, i.e., low pH leads to pale meat and color changes with reduced water-holding capacity, while higher pH leads to dark-colored meat, dryness, and poor storage quality [45]. The results of our study show that PJ supplementation (initially) resulted in lower temperature and pH but had no effect on breast meat color. In contrast, pH, yellowness, and the saturation index were lower 24 h after slaughter in breast meat from broilers receiving 9 g PJ/kg. The decrease in temperature and pH within 15 min after slaughter may be attributed to the bioactive compounds in the PJ powder that break down fat by activating the enzymes responsible for glycolysis. Decomposition may continue until after slaughter by reducing acidity and increasing carcass temperature, resulting in pale color. Another possibility is that the lower temperature of breast meat in chickens fed PJ may be due to the method of slaughter and preparation used (defeathering in a boiling tank at different temperatures). The results show that the color value of breast meat of broilers fed PJ did not change and was therefore within the normal range. This is consistent with studies by Tavaniello et al. [46] and Janocha et al. [47], who reported that the normal pH range for broiler meat is between 5.6 and 6.1. Tashla et al. [27] reported that the color becomes pale due to the low pH of the breast meat, which causes light scattering and reduces the degree of yellowness. However, a diet supplemented with a high level of PJ (9 g PJ/kg) decreased the pH, yellowness, and satiety index 24 h after slaughter.

Our findings indicate that the chemical properties of breast meat, such as moisture, crude protein, crude fat, ash, and organic matter, were not affected by the addition of PJ to the diet. One of the most crucial indicators of meat quality is the fatty acid composition of breast meat [48]. The levels of saturated fatty acids (3 g PJ) and monounsaturated fatty acids (3 and 9 g PJ) were higher, while the total levels of polyunsaturated fatty acids and the unsaturated-to-saturated fatty acids ratio (3 and 6 g PJ/kg) were lower. In contrast, the omega-6 fatty acids and omega-6 to omega-3 ratios decreased at levels of 3 g PJ/kg. Chicken meat contains a high percentage of unsaturated fatty acids, but polyunsaturated fatty acids are the most sensitive to oxidation processes and lipid oxidation in meat [49]. Therefore, a reduction in polyunsaturated fatty acids in breast meat of broilers fed PJ may improve meat quality and reduce oxidation. The reduced ratio of unsaturated to saturated fatty acids may indicate that PJ has antioxidant activity due to its contents of many bioactive compounds, such as flavonoids, phenols, and terpenoids. The polyunsaturated fatty acid content decreased in chickens fed 3 and 6 g PJ powder, which affected the total omega-6 fatty acid content and the ratio of omega-6 to omega-3 fatty acids in breast meat. This result may suggest that PJ has an effect on lipid metabolism due to its bioactive components, fatty acid profile, and the level of dietary supplementation with PJ powder.

Improving meat quality by focusing on nutrition and supplementation is a very important strategy for broiler production, the food industry, human consumption, and purchasing power [50]. Breast meat quality has been studied for its nutritional and commercial value in the broiler industry [51]. Savković et al. [52] reported that dietary supplementation with aromatic herbs had a positive effect on broiler meat quality. Our results show that broiler diets supplemented with PJ had a higher percentage of cooking water loss (6 and 9 g PJ/kg) and shear force (3 to 9 g PJ/kg), with no effect on the water-holding capacity and texture profile analysis parameters of breast meat, indicating that a diet supplemented with 3 g PJ may prevent breast muscle shrinkage during cooking, while a high level of PJ (6 and 9 g PJ/kg) increases the percentage of water loss during cooking, which may be directly related to the loss of juiciness due to the chemical properties of this aromatic medicinal plant [53]. The diet supplemented with PJ contributed to a significant increase in breast meat shear force in chickens fed 3 to 9 g PJ, which may be due to an increase in muscle fiber [54]. This can be explained by the influence of the active ingredients in PJ (phenols and flavonoids) on fat metabolism and the highest utilization from ingested feed.

## 5. Conclusions

The results indicate that growth performance parameters (body weight, weight gain, feed conversion ratio, and production efficiency index) improve when broilers are fed PJ, especially in the finishing phase. In addition, carcass characteristics and some meat quality parameters were generally improved by PJ supplementation. The addition of PJ to the birds’ diets also enhanced their fatty acid profiles. Overall, the inclusion of PJ in broiler diets had a positive effect on most carcass and meat quality indices. Therefore, it is strongly recommended that further studies be conducted to validate the potential applications of PJ in improving broiler performance and meat quality.

## Figures and Tables

**Table 1:** Feed ingredients and calculated nutrients of basal diet.

Feed Ingredient (g/kg)	Feeding Stages		
	Starter 1–10 Days Old	Grower 11–24 Days Old	Finisher 25–36 Days Old
Yellow corn	526	573	613
Soybean meal (48%)	392	341	292
Corn oil	37	44	53
Di-calcium phosphate	18	16	15
Limestone	10	9.2	8.6
Sodium chloride	4.2	3.2	3.3
DL-methionine	3.5	3.2	2.9
L-lysine hydrochloride	2.0	1.9	1.9
L-threonine	1.3	1.1	0.9
Vit. and min premix *	5.0	5.0	5.0
Choline CL (60%)	0.9	0.9	1.0
Sodium bicarbonate	0.1	1.5	3.4
Calculated nutrients (g/kg)			
Metabolizable energy (ME; kcal)	3000	3100	3200
Crude protein	232.9	211.5	190.9
Crude fat	65.1	72.6	81.6
Crude fiber	28.3	27.2	26.1
Calcium	9.6	8.7	7.9
Available phosphorus	4.8	4.4	4.0
Digestible lysine	12.8	11.5	10.3
Digestible methionine + cysteine	9.5	8.7	8.0
Digestible threonine	8.6	7.7	6.9
Digestible arginine	14.3	12.8	11.4

* Dietary content per kilogram of premix: vitamin A = 2,400,000 IU; vitamin B1 = 600 mg; vitamin B2 = 1600 mg; vitamin B6 = 1000 mg; vitamin B12 = 6 mg; biotin = 40 mg; niacin = 8000 mg; folic acid = 400 mg; vitamin D = 1,000,000 IU; vitamin E = 16,000 IU; vitamin K = 800 mg; pantothenic acid = 3000 mg; cobalt = 80 mg; copper = 2000 mg; iodine = 400 mg; zinc = 14,000 mg; iron = 1200 mg; manganese = 18,000 mg; selenium = 60 mg.

**Table 2:** Growth performance indicators in broilers fed diets supplemented with *Pulicaria jaubertii* (PJ).

	PJ Levels					
Parameter	0 g/kg	3 g/kg	6 g/kg	9 g/kg	SEM ^1^	*p-*Value
Starter stage (1–10 d)						
Body weight, g, 1 d	46.62	46.62	46.60	46.64	0.04	0.079
Body weight, g, 10 d	258.6 ^b^	280.0 ^a^	273.7 ^a^	274.6 ^a^	4.42	0.020
Weight gain, g	214.7	233.5	227.2	228.1	4.42	0.057
Feed intake, g	260.3	269.1	264.3	265.7	3.32	0.413
Feed conversion ratio, g:g	1.21	1.15	1.17	1.17	0.02	0.148
Production efficiency index	213.9 ^b^	243.5 ^a^	235.7 ^ab^	235.6 ^ab^	6.76	0.041
Grower stage (11–24 d)						
Body weight, g, 24 d	1161.7	1171.4	1137.1	1180.9	33.62	0.815
Weight gain, g	888.5	882.6	852.0	898.8	26.99	0.768
Feed intake, g	1263.5	1198.1	1158.8	1211.7	30.48	0.193
Feed conversion ratio, g/g	1.42 ^a^	1.36 ^ab^	1.37 ^ab^	1.34 ^b^	0.02	0.048
Production efficiency index	340.8	359.8	348.3	365.2	11.43	0.534
Finisher stage (25–36 d)						
Body weight, g, 36 d	2321.6 ^b^	2447.9 ^a^	2481.6 ^a^	2469.0 ^a^	31.52	0.008
Weight gain, g	1111.3 ^b^	1245.5 ^a^	1216.5 ^a^	1240.5 ^a^	22.96	<0.0001
Feed intake, g	1758.0	1676.1	1955.0	1711.5	100.6	0.395
Feed conversion ratio, g/g	1.75 ^a^	1.46 ^b^	1.52 ^b^	1.50 ^b^	0.03	<0.0001
Production efficiency index	419.7 ^b^	519.9 ^a^	508.3 ^a^	511.5 ^a^	10.01	<0.0001

^a,b^ Expression of significant difference (*p* < 0.05) is indicated by superscript numbers above the means (*n* = 10 replicate cages per PJ level) of each parameter in the row. ^1^ SEM = standard error of means.

**Table 3:** Carcass traits and body components in broilers fed diets supplemented with *Pulicaria jaubertii* (PJ).

	PJ Levels					
Parameter	0 g/kg	3 g/kg	6 g/kg	9 g/kg	SEM ^1^	*p-*Value
Preslaughter weight, g	2093 ^b^	2536 ^a^	2644 ^a^	2475 ^a^	77.3	0.002
Hot carcass weight, g	1375 ^b^	1632 ^a^	1737 ^a^	1653 ^a^	50.1	0.002
Cold carcass weight, g	1327 ^b^	1575 ^a^	1666 ^a^	1561 ^a^	48.9	0.004
Dressing yield, %	65.67	64.35	65.78	66.83	0.63	0.076
Legs, %	37.59 ^a^	36.43 ^ab^	34.92 ^b^	35.48 ^b^	0.61	0.027
Breast, %	38.17	41.19	40.52	37.89	1.05	0.085
Back, %	8.16 ^a^	7.24 ^b^	7.33 ^b^	6.79 ^b^	0.28	0.016
Abdominal fat, %	0.89 ^ab^	1.09 ^a^	0.78 ^ab^	0.67 ^b^	0.12	0.012

^a,b^ Expression of significant difference (*p* < 0.05) is indicated by superscript numbers above the means (*n* = 10 replicate chickens per PJ level) of each parameter in the row. ^1^ SEM = standard error of means.

**Table 4:** Physical properties of breast meat in broilers fed diets supplemented with *Pulicaria jaubertii* (PJ).

	PJ Levels					
Parameter	0 g/kg	3 g/kg	6 g/kg	9 g/kg	SEM ^1^	*p-*Value
Initial—15 min post slaughter						
Temp., °C	32.57 ^a^	30.81 ^b^	30.45 ^b^	30.36 ^b^	0.32	<0.0001
pHi	6.27 ^a^	6.11 ^b^	6.17 ^b^	6.17 ^b^	0.03	0.007
Color values						
Lightness	47.90	47.50	47.19	46.94	0.64	0.751
Redness	0.30	0.84	0.53	1.07	0.45	0.645
Yellowness	10.51	9.26	9.14	9.05	0.50	0.154
Color change	46.77	47.03	47.33	47.57	0.64	0.831
Saturation index	10.59	9.40	9.32	9.16	0.50	0.179
Hue angle	34.60	30.30	14.13	65.13	23.95	0.507
Browning index	24.85	22.55	21.95	22.93	1.52	0.575
Ultimate—24 h post slaughter						
pHu	6.04 ^a^	6.04 ^a^	6.00 ^ab^	5.95 ^b^	0.02	0.008
pH different	0.23 ^a^	0.07 ^b^	0.17 ^a^	0.21 ^a^	0.03	0.004
Color values						
Lightness	47.78	46.48	47.52	45.16	0.78	0.093
Redness	2.73	3.08	4.04	3.82	0.57	0.341
Yellowness	16.25 ^a^	15.33 ^a^	15.59 ^a^	13.16 ^b^	0.71	0.024
Color change	48.21	49.22	48.36	50.12	0.72	0.235
Saturation index	16.63 ^a^	15.72 ^ab^	16.22 ^a^	13.76 ^b^	0.68	0.027
Hue angle	80.19	78.52	75.33	73.44	2.23	0.153
Browning index	45.19	44.17	45.38	40.41	2.25	0.382

^a,b^ Expression of significant difference (*p* < 0.05) is indicated by superscript numbers above the means (*n* = 10 replicate chickens per PJ level) of each parameter in the row. ^1^ SEM = standard error of means.

**Table 5:** Chemical properties of breast meat in broilers fed diets supplemented with *Pulicaria jaubertii* (PJ).

	PJ Levels					
Parameter (%)	0 g/kg	3 g/kg	6 g/kg	9 g/kg	SEM ^2^	*p-*Value
Moisture	73.08	74.04	73.90	73.16	0.36	0.185
Crude protein	22.98	22.35	22.68	23.27	0.39	0.411
Crude fat	2.64	2.23	2.45	2.23	0.32	0.780
Ash	1.29	1.37	1.35	1.33	0.04	0.420
Organic matter ^1^	98.71	98.62	98.65	98.67	0.04	0.421
Fatty acid profiles						
Saturated fatty acids	36.01 ^bc^	38.57 ^a^	37.85 ^ab^	33.86 ^c^	0.70	0.006
Monounsaturated fatty acids	34.79 ^b^	37.87 ^a^	36.07 ^ab^	37.90 ^a^	0.65	0.026
Polyunsaturated fatty acids	29.19 ^a^	23.56 ^c^	26.07 ^bc^	28.23 ^ab^	0.86	0.007
Omega-6 fatty acids	28.15 ^a^	23.57 ^b^	26.62 ^a^	28.30 ^a^	0.89	0.018
Omega-3 fatty acids	0.71	0.73	0.71	0.66	0.03	0.374
Omega-6/omega-3	39.70 ^ab^	32.38 ^c^	37.38 ^b^	42.98 ^a^	1.28	0.002
Unsaturated/saturated fatty acids	1.78 ^a^	1.59 ^b^	1.64 ^b^	1.95 ^a^	0.04	0.006

^a,b,c^ Expression of significant difference (*p* < 0.05) is indicated by superscript numbers above the means (*n* = 10 replicate chickens per PJ level) of each parameter in the row. ^1^ Organic matter = 100-Ash. ^2^ SEM = standard error of means.

**Table 6:** Breast meat quality parameters in broilers fed diets supplemented with *Pulicaria jaubertii* (PJ).

	PJ Levels					
Parameter	0 g/kg	3 g/kg	6 g/kg	9 g/kg	SEM ^1^	*p-*Value
Water-holding capacity (%)	28.83	28.48	28.29	26.85	0.81	0.349
Cooking water loss (%)	9.54 ^b^	9.70 ^b^	13.36 ^a^	14.63 ^a^	1.47	0.041
Shear force (kg/cm^2^)	6.61 ^b^	10.20 ^a^	10.08 ^a^	10.60 ^a^	0.36	0.001
Texture profile analysis						
Hardness	13.99	12.88	14.51	10.42	1.22	0.102
Springiness	0.64	0.63	0.60	0.60	0.02	0.411
Cohesiveness	0.36	0.38	0.36	0.35	0.01	0.455
Gumminess	5.16	5.08	5.35	3.70	0.52	0.118
Chewiness	3.28	3.29	3.14	2.20	0.31	0.051
Resilience	0.15	0.16	0.16	0.17	0.01	0.525

^a,b^ Expression of significant difference (*p* < 0.05) is indicated by superscript numbers above the means (*n* = 10 replicate chickens per PJ level) of each parameter in the row. ^1^ SEM = standard error of means.

## Data Availability

All data in this study are available from the first author (H.H. Al-Baadani).

## Supplementary Materials

The following supporting information can be downloaded at: https://www.mdpi.com/article/10.3390/life13081780/s1, Table S1: Breast meat fatty acid profile in broilers fed diets supplemented with *Pulicaria jaubertii* (PJ).
