# Supplementary material for: Evaluation of Carcass Attributes and Physical, Chemical, and Qualitative Characteristics of Breast Meat of Broiler Chickens Fed on Pulicaria jaubertii Powder
Source: Life (Basel). 2023 Aug 21;13(8):1780. doi: 10.3390/life13081780 (PMC10455954; doi:10.3390/life13081780)
Supplement: Supplementary file 1 [file life-13-01780-s001.zip › life-2521184-supplementary.pdf]

Supplementary File

Table S1. Breast meat fatty acid profile in broilers fed diets supplemented with *Pulicaria jaubertii* (PJ).

**Parameters**	**PJ levels**				SEM[1]	***P-value***
	0 g / kg	3 g / kg	6 g / kg	9 g / kg		
			Saturated fatty acids			
Capric acid	0.18	0.27	0.27	0.25	0.05	0.399
Lauric acid	0.35	0.31	0.29	0.36	0.06	0.742
Myristic acid	2.90	2.85	2.57	2.03	0.61	0.710
Pentadecanoic acid	1.13	1.74	1.35	1.07	0.21	0.066
Palmitic acid	20.62	21.74	21.26	21.11	0.50	0.519
Heptadecanoic acid	0.28	0.45	0.30	0.39	0.05	0.167
Stearic acid	6.20	7.22	6.46	6.75	0.30	0.176
Pristanic acid	0.23	0.20	0.25	0.23	0.01	0.107
Lignoceric acid	3.27	4.91	4.91	4.71	1.03	0.666
			Monounsaturated fatty acids			
Tetradecenoic acid	0.73	0.70	0.72	0.61	0.08	0.123
Myristioleic acid	1.28	1.15	1.17	1.60	0.35	0.846
Palmitoleic acid	1.56	2.58	2.66	2.27	0.63	0.611
Vaccinic acid	1.56	1.54	1.44	1.37	0.08	0.458
Oleic acid	30.52	30.55	34.04	34.47	1.32	0.948
Gondoic acid	0.20	0.32	0.34	0.21	0.05	0.180
			Polyunsaturated fatty acids			
Linoleic acid	25.70	20.11	23.41	25.07	1.15	0.284
α-Linolenic acid	0.71	0.73	0.71	0.63	0.04	0.284
Y-Linolenic acid	0.18	0.12	0.19	0.19	0.03	0.174
Eicosadienoic acid	0.27	0.36	0.37	0.36	0.07	0.752
Eicosatrienoic acid	0.25	0.30	0.36	0.27	0.08	0.807
Arachidonic acid	2.01	1.59	1.41	1.53	0.33	0.615
Docosatetraenoic acid	0.41	0.40	0.61	0.47	0.14	0.797

[a-b] Expression of significant difference ($P < 0.05$) is indicated by superscript numbers above the means (n= 10 replicate chickens per PJ level) of each parameter in the row. [1] SEM= Standard error of means.
